# Visual Working Memory Capacity Does Not Modulate the Feature-Based Information Filtering in Visual Working Memory

**DOI:** 10.1371/journal.pone.0023873

**Published:** 2011-09-16

**Authors:** Jifan Zhou, Jun Yin, Tong Chen, Xiaowei Ding, Zaifeng Gao, Mowei Shen

**Affiliations:** Department of Psychology and Behavioral Sciences, Zhejiang University, Hangzhou, China; Kyushu University, Japan

## Abstract

**Background:**

The limited capacity of visual working memory (VWM) requires us to select the task relevant information and filter out the irrelevant information efficiently. Previous studies showed that the individual differences in VWM capacity dramatically influenced the way we filtered out the distracters displayed in distinct spatial-locations: low-capacity individuals were poorer at filtering them out than the high-capacity ones. However, when the target and distracting information pertain to the same object (i.e., multiple-featured object), whether the VWM capacity modulates the feature-based filtering remains unknown.

**Methodology/Principal Findings:**

We explored this issue mainly based on one of our recent studies, in which we asked the participants to remember three colors of colored-shapes or colored-landolt-Cs while using two types of task irrelevant information. We found that the irrelevant high-discriminable information could not be filtered out during the extraction of VWM but the irrelevant fine-grained information could be. We added 8 extra participants to the original 16 participants and then split the overall 24 participants into low- and high-VWM capacity groups. We found that regardless of the VWM capacity, the irrelevant high-discriminable information was selected into VWM, whereas the irrelevant fine-grained information was filtered out. The latter finding was further corroborated in a second experiment in which the participants were required to remember one colored-landolt-C and a more strict control was exerted over the VWM capacity.

**Conclusions/Significance:**

We conclude that VWM capacity did not modulate the feature-based filtering in VWM.

## Introduction

Visual working memory (VWM) is one of the most critical modules in our information processing system, yet it only maintains a very limited amount of information [1], [2], [3], [4], [5]. For instance, only at most 3∼4 simple objects could be retained in VWM at a time [1], [6], [7], [8], [9], [10]. This limit-capacity requires that while efficiently selecting the task relevant information, we should also filter out the task irrelevant information [11]. Recently a few studies have been conducted on the filtering of the irrelevant information, particularly when multiple visual objects occupied distinct spatial locations (i.e., location-based filtering) [12], [13], [14]. However, when multiple features share the same spatial location in an object (e.g., colored shapes), how VWM filters the irrelevant information (i.e., feature-based filtering) remains largely unclear. Here we explored this issue by focusing on whether the feature-based filtering mechanism was modulated by VMW capacity.

In what could be considered as a seminar work, Vogel and colleagues explored how the participants selected information in a set of visual objects containing both targets and distracters [12], which were presented in multiple locations separately (note although in Experiment 1 of [12], the participants were asked to select the objects based on color and Vogel and colleagues considered it as feature-based, spatial location information of color was still needed to select the color and hence it was location-based in our definition). They found that the high-capacity group and the low-capacity group exhibited distinct filtering mechanisms: while the high-capacity group entailed an excellent top-down control by predominantly allowing the targets to access to VWM and filtering the distracters efficiently, the low-capacity group exhibited a poor filtering ability as the distracters were not filtered out but instead extracted into VWM. Supporting these findings, they also found a significant positive correlation between VWM capacity and the filtering efficiency. In line with the suggestion that the low-capacity group had a loose top-down control such that the task-irrelevant information was also selected, a verbal working memory study revealed that comparing to the high-capacity group, the low-capacity group was more likely to notice their own names among those unattended, irrelevant messages (i.e., cocktail party phenomenon) [15]. Realizing these converging evidences on the filtering of the incoming irrelevant information, it is thus possible that the VWM capacity also modulated the feature-based filtering in VWM in a similar fashion as mentioned above.

One of our recent studies provided us a good base to examine this possibility. In that study, we adopted two types of task-irrelevant information to investigate which could be selected into VWM involuntarily: high-discriminable information which is processed at the parallel stage of visual perception, and fine-grained information which is processed via focal attention [16]. By requiring the participants to remember one or three colored objects, we consistently found that there are dissociated extracting (or on the other side, filtering) mechanisms for the two types of information. Specifically, whereas the task-irrelevant high-discriminable information could not be filtered out but was selected into VWM regardless of the task demand, the irrelevant fine-grained information was successfully filtered out of the VWM. Since the materials were double-featured objects (e.g., colored shapes) while one was the task irrelevant dimension, this setting thus enabled us to explore two issues conveniently. First, whether the automatic selection of the irrelevant high-discrimination information was constrained to the low-capacity group; second, whether the filtering out of the irrelevant fine-grained information was predominantly restricted to the high-capacity group. The answers of them will also help us examine the stability of our previous findings [16].

To answer the above questions, we added 8 new participants to the previous 16 participants of Experiment 4 of Gao et al. (2010) [16], which allowed us to have a sound number of participants (i.e., 12 participants) in each capacity group. There are at least 2 reasons for us to set our investigation on this experiment. First, contrast to remembering 1 object in most of the experiments in Gao et al. [16], participants were required to remember 3 double-featured objects which is close to or reaches the ceiling of VWM capacity, avoiding the possible ceiling effect on the behavioral performance. This enabled us to split the participants into two groups based on the behavioral performance. Second, instead of presenting the two types of irrelevant information in different blocks, we displayed the two types of irrelevant information randomly while the same relevant dimension was remembered, which avoided the contamination of possible strategies used for the two types of irrelevant information.

To reiterate our previous logic [16], in a change detection task we manipulated the consistency of the irrelevant feature between the memory array and the test array. We asked the participants to pay attention to the relevant feature while ignoring the irrelevant feature. If the irrelevant feature could be selected into VWM, then its change in the test array would affect the behavioral performance of the target feature [17], [18], [19] and evoke a negative ERP component N270 in the frontal region compared to the no-change condition. The N270 is suggested to reflect the detailed comparison of individual features between the representation in VWM and the incoming perceptual input [16], [20], [21].

We predicted that if VWM capacity modulated the feature-based filtering in a similar way as that for the location-based filtering, then only the low-capacity group could encode the irrelevant information, whereas the high-capacity group would filter it out. In addition, if the feature-based filtering was further modulated by the *irrelevant information* type, then the low-capacity group may be only able to filter out the fine-grained information, but not the high-discriminable information since multiple lines of evidences showed that the latter could be involuntarily selected into VWM [16], [17], [20], [22], [23]. Furthermore, if the VWM capacity modulated the feature-based filtering as in the location-based filtering, we may also find a significant negative correlation (i.e., lower capacity participants exhibiting a higher N270) between the irrelevant-change-related N270 amplitude and the VWM capacity. To preview the results, we replicated our previous findings [16] in both low- and high-capacity groups and found a non-significant correlation between N270 amplitude and VWM capacity, suggesting that the VWM capacity did not modulate the feature-based filtering in VWM. Because of a null-effect revealed for the VWM capacity over the feature-based filtering, we further conducted a second experiment using a one-item load condition and had a more strict control over the VWM capacity. The results further corroborated our findings.

## Experiment 1

### Methods

#### Participants

We added 8 new participants into the previous 16 participants, which resulted in 24 right-handed students (13 females; mean age 23.8 years) which were recruited from Zhejiang University. All the participants had normal or corrected-to-normal vision and no history of neurological problems. All participants provided written and informed consent before experiments, and all procedures were approved by the Research Ethics Board of Zhejiang University.

For each participant, we first estimated the VWM capacity by using Cowan's *K* formula [24]: *K* = *S* * (*H*−*F*), where *K* is the VWM capacity, *S* is the number of displayed objects, *H* is the hit rate, and *F* is the false alarm rate. Since the overall performance of detecting color-change was equal between colored shapes and colored landolt-Cs [*F*<1], we hence calculated the K by pooling the two conditions together. Participants were then divided into high-capacity group (K = 2.8) and low-capacity group (K = 2.5) using a median split of their VWM capacity estimates, resulting in 12 participants in each group.

#### Stimuli

**Figure 1:**
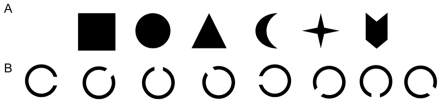
Example of materials used in Experiment 1. (A) Objects containing high-discriminable information (shape). (B) Objects containing fine-grained information (gap orientation).

Colored landolt-Cs (Figure 1A, 1.01°×1.01° of visual angle for the circle, 0.25° of visual angle for the gap's height from a viewing distance of 70 cm) were employed for the fine-grained condition, and a group of colored shapes (about 1.01°×1.01° of visual angle) were used for the high-discriminable condition (Figure 1B). The orientation of a landolt-C was selected from a set of 8 orientations: 0°, 45°, 90°, 135°, 180°, 225°, 270° and 315°. The shape was selected from a set of 6 distinct shapes. Seven colors were adopted: red (255, 0, 0 in RGB value), green (0, 255, 0), blue (0, 0, 255), violet (255, 0, 255), yellow (255, 255, 0), black (0, 0, 0), and white (255, 255, 255). The stimuli were presented at the center of a 17 inch monitor with grey (128, 128, 128) background.

#### Procedure and Design

**Figure 2:**
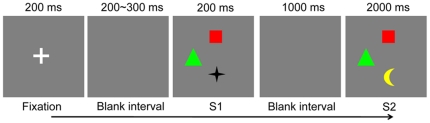
Illustration of a trial in Experiment 1, in which the black star is changed to a yellow moon.

The procedure of the experiment was illustrated in Figure 2. After a variable delay ranging from 1000 to 1400 ms, a fixation was displayed for 200 ms followed by a 200∼300 ms blank interval. Then Stimulus Set 1 (S1) was displayed for 200 ms, followed by a 1000 ms blank period. Finally Stimulus Set 2 (S2) was always presented for 2000 ms regardless of whether the participant made a response or not, which was used to exclude any possible contamination of ERP response caused by the offset of S2. Both S1 and S2 contained 3 colored objects, which were displayed randomly in three of four possible locations with a distance of 1.8° visual degree to the center of screen. For the four locations, two of them were located horizontally while the other two vertically. In 50% of trials, S1 and S2 were colored shapes; while in the other 50% trials, they were colored landolt-Cs. Participants were instructed to remember the colors of the three objects while ignoring the irrelevant shapes or orientations, and justify whether the colors of S2 were identical to S1. Only the response accuracy was emphasized and recorded in the current experiment.

The four change types were: S2 was either the same as S1 (*no change*), or different from S1 in one shape or three orientations (*irrelevant change*), or different from S1 in one color (*relevant change*), or S1 and S2 different in one color and one shape (belonging to the same object) or one color and three orientations (*both change*) (please see Experiment 4 of [16] for detailed explanation of this setting). The change of three orientations was used to avoid the caveat that the non-influence of fine-grained information change was due to its weak signal of change.

A 2 (Irrelevant-type: shape, orientation)×4 (Change-type: no change, irrelevant change, relevant change, and both change) within-subject design was adopted. Participants completed 80 trials under each of the 8 conditions, resulting in a total of 640 trials which were presented randomly. The experiment was divided into 8 blocks with 5-minute break in between, which lasted about 1 hour in total.

#### Electrophysiological Recording and Analysis

The EEG was recorded from 32 scalp sites using Ag/AgCl electrodes mounted in an elastic cap. All recordings were initially referenced to the left mastoid, and re-referenced offline to the average of the left and right mastoids. Vertical electrooculogram (VEOG) and horizontal electrooculogram (HEOG) were recorded with two pairs of electrodes, one pair placed above and below the left eye, and the other pair placed beside the two eyes. All interelectrode impedances were maintained below 5 ΚΩ. The EEG and EOG were amplified by SynAmps using a 0.05–100 Hz bandpass and continuously sampled at 1000 Hz/channel for off-line analysis. Electrooculogram artifacts were corrected using a regression method [25]. Additional artifact rejection was applied to epochs with EEG amplitude exceeding ±75 µV. An average of 12.1% of trials for shape as the irrelevant dimension and an average of 12.4% of trials for orientation as the irrelevant dimension were excluded from further ERP analysis. The EEG and EOG were digitally filtered offline with a 0.05–30 Hz bandpass filter. The EEG was segmented into 2100-ms epochs starting from 100 ms before S1 onset, which was used as the baseline.

Based on previous studies [16], [20], [26], [27], [28], [29], [30] and scrutiny of the present N270 distribution, the statistical analysis was mainly restricted to the frontal regions. Since the ERP waveforms were fairly similar across the frontal electrodes (FP1, FP2, FCZ, FZ, F3, F4, FC3, and FC4) and our previous studies did not reveal an significant interaction between change types and electrodes [16], [20], [31], we thus averaged these electrodes to form one representative electrode, which would help us focus on the change-related signals. The averaged amplitudes of N270 of the selected time window (details see below) were put into statistics. A mixed analysis of variance (ANOVA) by taking Change-type (no change, irrelevant change, relevant change, and both change) as the within-subject factor and Group (low-capacity versus high-capacity) as the between-subject factor was conducted separately for colored shapes and landolt-Cs. Greenhouse-Geisser correction was adopted when necessary. Planned contrast was conducted by comparing irrelevant change, relevant change, and both change with no change, to evaluate N270 and the influence of mismatch on accuracy in each capacity group. For N270, only the correct-response trials were analyzed. Finally, we calculated the Pearson correlation between the irrelevant-change-related N270 amplitude and the VWM capacity. To obtain pure ERP activities related to the irrelevant change, a difference wave was constructed by subtracting the no change from the irrelevant change. The amplitude of a time window, which was taken for measuring the N270 in the original ERP, was used in the difference wave for the correlation calculation.

### Results and Discussion for High-discriminable Shape as Irrelevant Feature

#### Behavioral Data

**Figure 3:**
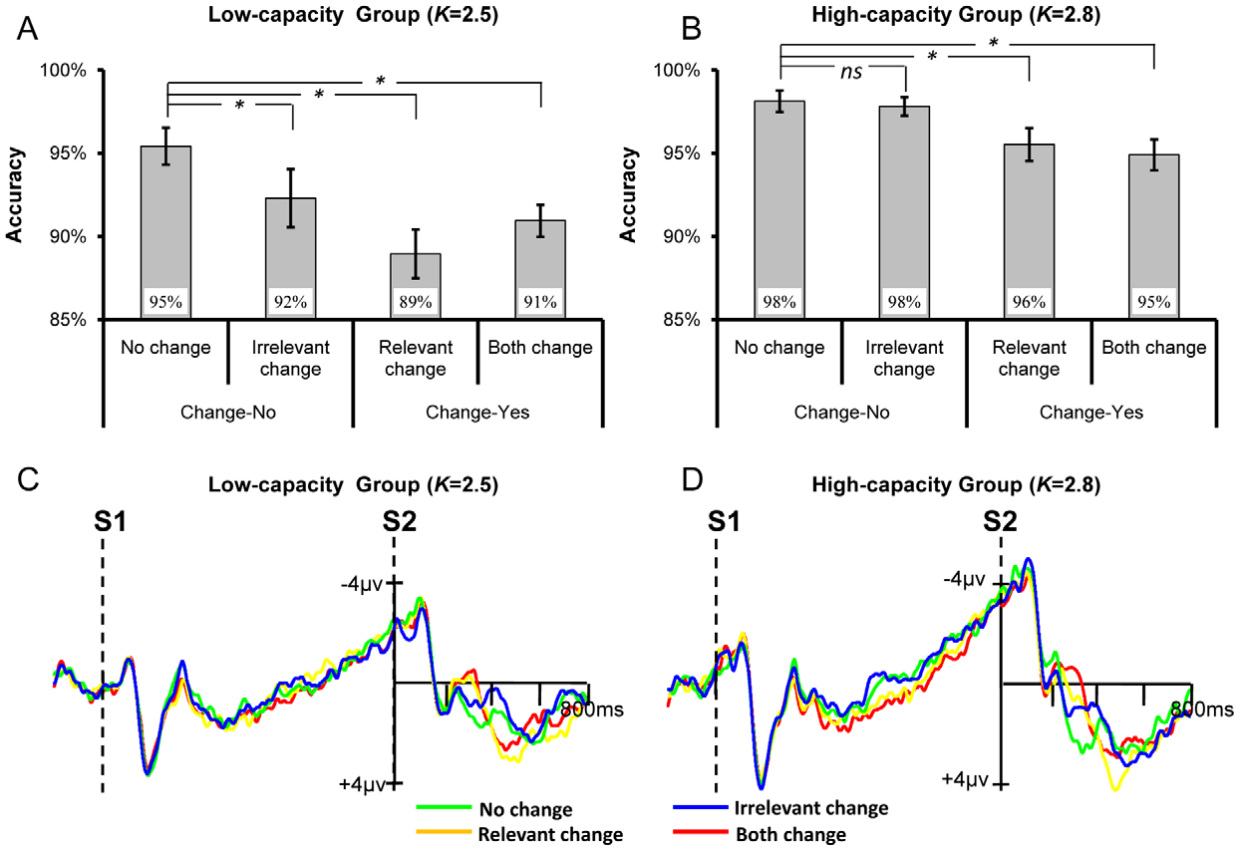
The results for the low- and high-capacity groups when shape was the task-irrelevant information. The top row shows the accuracy for the low-capacity group (A) and the high-capacity group (B), respectively. The bottom row shows the ERP results for the low-capacity group (C) and the high-capacity group (D), respectively.

The behavioral results were shown in Figure 3A and 3B. The mixed ANOVA showed that the accuracy was considerably higher in the high-capacity group (97%) than in the low-capacity group [92%; *F*(1,22) = 19.19, *p*<0.001, partial*η^2^* = 0.47, *ω* = 0.99]. It also revealed a significant main effect of Change-type [*F*(3,66) = 9.62, *p* = 0.001, partial*η^2^* = 0.30, *ω* = 0.96], yet the Change-type×Group interaction was not significant [*F*(3,66) = 1.62, *p* = 0.19, partial*η^2^* = 0.07, *ω* = 0.41]. Planned contrast based on all participants showed that the accuracy of irrelevant change (95%) was significantly lower than no change [97%; *F*(1,23) = 5.94, *p*<0.025, partial*η^2^* = 0.21, *ω* = 0.65]. This result suggested that the irrelevant shape was encoded into VWM and impaired the detection of colors. Both the accuracy of relevant change [92%; *F*(1,23) = 15.12, *p* = 0.001, partial*η^2^* = 0.40, *ω* = 0.96] and both change (93%) were also lower than no change [97%; *F*(1,23) = 37.53, *p*<0.001, partial*η^2^* = 0.62, *ω* = 1.0]. No difference was found between relevant change (92%) and both change [93%; *F*<1]. These results replicated our previous findings [16].

We then conducted planned contrasts within each capacity group separately to re-verify the above non-significant Change-type×Group interaction. It showed that whereas for the low-capacity group (see Figure 3A) irrelevant change significantly impaired the participants' performance relative to no change [*F*(1,11) = 8.33, *p* = 0.015, partial*η^2^* = 0.43, *ω* = 0.75], for the high-capacity group (see Figure 3B) there was no difference between irrelevant change and no change [*F*<1, *p* = 0.68, partial*η^2^* = 0.02, *ω* = 0.07]. For both groups the accuracy of relevant change and both change were significantly lower than no change [all *p*s<0.05, partial*η^2^*>0.35, *ω*>0.55], while no difference was found between the two [both *p*s>0.2].

#### ERP Data

As evident in Figure 3C and 3D, in both groups the change of irrelevant shape elicited N270. Following the same way we adopted before [16], while a window of 350–410 ms was adopted to test the effect of irrelevant shape change because of a delay of N270, for the other three conditions a time window of 260–380 ms was used to measure the change effect. A mixed ANOVA was thus run separately for the two different time windows.

We first ran a mixed ANOVA by taking the Change-type (no change versus irrelevant change) as the within-subject factor and Group as the between-subject factor to examine the effect of irrelevant change. It revealed a significant main effect of Change-type [*F*(1,22) = 16.46, *p* = 0.001, partial*η^2^* = 0.43, *ω* = 0.97], yet neither the main effect of Group [*F*(1,22)<1, *p*>0.40, partial*η^2^* = 0.02, *ω* = 0.11] nor the Group×Change-type interaction [*F*(1,22)<1, *p*>0.40, partial*η^2^* = 0.02, *ω* = 0.11] was significant. These results suggested that a significant N270 was evoked by the irrelevant change, and it was not modulated by the VWM capacity.

A second mixed ANOVA by taking Change-type (no change, relevant change, and both change) as the within-subject factor and Group as the between-subject factor also revealed a significant main effect of Change-type [*F*(2,44) = 17.25, *p*<0.001, partial*η^2^* = 0.44, *ω* = 1.0], however, it was not modulated by the Group [Group×Change-type interaction: *F*(2,44) = 1.15, *p*>0.30, partial*η^2^* = 0.05, *ω* = 0.24]. The main effect of Group was not significant [*F*(1,22) = 1.86, *p*>0.10, partial*η^2^* = 0.08, *ω* = 0.26], either.

Planned contrast further confirmed the above findings. A significant larger negativity was elicited by the irrelevant change relative to the no change for both low-capacity group [*F*(1,11) = 5.89, *p*<0.05, partial*η^2^* = 0.35, *ω* = 0.60] and high-capacity group [*F*(1,11) = 10.83, *p*<0.01, partial*η^2^* = 0.50, *ω* = 0.85]. Additionally, in both groups relevant change and both change evoked a significant N270 than no change [both *p*s<0.05, partial *η^2^*>0.40, *ω*>0.70].

#### Correlation

**Figure 4:**
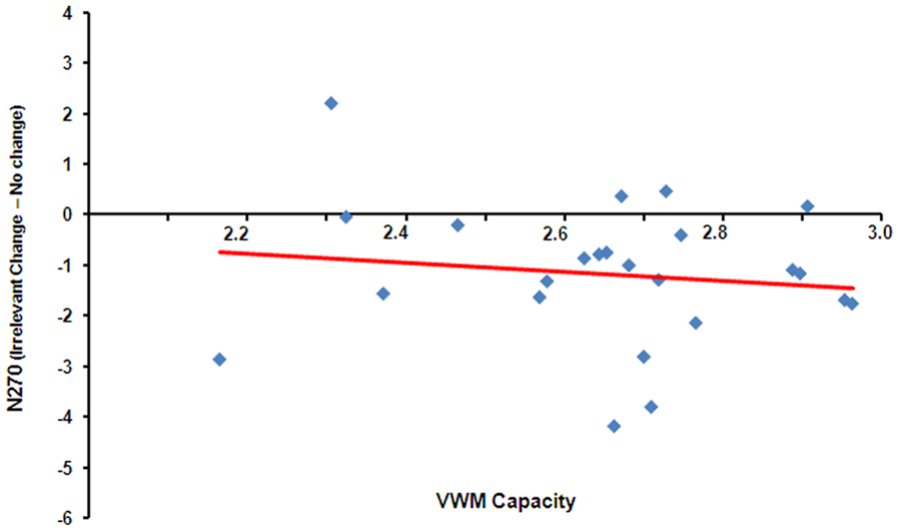
The correlation between individual VWM capacity and the corresponding N270 amplitude of the irrelevant change. The red line is the linear regression line fitting the data points.

As shown in Figure 4, the correlation between the VWM capacity and N270 amplitude was very weak [Pearson *r* = −0.013, *p* = 0.55].

#### Interim Discussion

Overall, both the behavioral and the ERP results replicated our previous findings [16]. More importantly, although we did not find an effect of the irrelevant change in the high-capacity group on accuracy, which may be due to the ceiling effect of the performance, in both capacity groups we consistently found the ERP evidence suggesting that the irrelevant high-discriminable feature was encoded into VWM and its change evoked a significant N270. This finding was further confirmed by the non-significant correlation between the N270 amplitude evoked by the irrelevant change and the VWM capacity.

### Results and Discussion for Fine-grained Orientation as Irrelevant Feature

#### Behavioral Data

**Figure 5:**
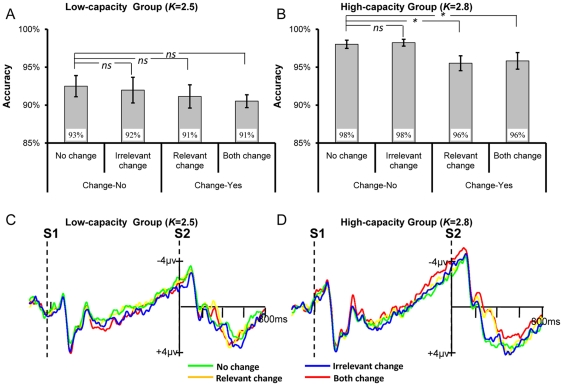
The results for the low- and high-capacity groups when gap orientation was the task-irrelevant information. The top row shows the accuracy for the low-capacity group (A) and the high-capacity group (B), respectively. The bottom row shows the ERP results for the low-capacity group (C) and the high-capacity group (D), respectively.

The behavioral results were shown in Figure 5A and 5B. The mixed ANOVA revealed that the accuracy was significantly higher in the high-capacity group (97%) than in the low-capacity group [92%; *F*(1,22) = 26.25, *p*<0.001, partial*η^2^* = 0.54, *ω* = 1.0]. The main effect of Change-type was not significant [*F*(3,66) = 2.43, *p*>0.1, partial*η^2^* = 0.1, *ω* = 0.47], neither was the Group×Change-type interaction [*F*(3,66)<1, *p*>0.8, partial*η^2^* = 0.01, *ω* = 0.10]. The followed-up planned contrast revealed that no difference existed between irrelevant change (95%) and no change [95%; *F*(1,23)<1, *p*>0.7, partial*η^2^* = 0, *ω* = 0.06]. The accuracy of relevant change (93%) was marginally lower than no change [95%; *F*(1,23) = 3.38, *p* = 0.08, partial*η^2^* = 0.13, *ω* = 0.42], while both change (93%) was significantly lower than no change [95%; *F*(1,23) = 4.49, *p*<0.05, partial*η^2^* = 0.16, *ω* = 0.53]. There was no difference between relevant change and both change [*F*(1,23)<1, *p*>0.8, partial*η^2^* = 0, *ω* = 0.05]. Again, these results replicated our previous findings [16].

We then conducted the planned contrasts within both capacity groups separately. It showed that there was no difference at all between irrelevant change and no change for both low-capacity group (Figure 5A) [*F*(1,11)<1, *p*>0.5, partial*η^2^* = 0.03, *ω* = 0.09] and high-capacity group (Figure 5B) [*F*(1,11)<1, *p*>0.7, partial*η^2^* = 0.01, *ω* = 0.06]. Besides, the accuracy of relevant change [*F*(1,11) = 10.15, *p*<0.01, partial*η^2^* = 0.48, *ω* = 0.83] and both change [*F*(1,11) = 6.49, *p*<0.05, partial*η^2^* = 0.37, *ω* = 0.64] were significantly lower than no change for the high-capacity group, yet these effects vanished in the low-capacity group [both *p*s>0.1]. No difference was found between relevant change and both change for both groups [both *p*s>0.5].

#### ERP Data

Replicating our previous findings [16], in both groups the change of irrelevant fine-grained feature did not evoke a larger negativity during the time window of N270 (Figure 5C & 5D). In addition, the N270 evoked by relevant change and both change were more prominent in the high-capacity group than in the low-capacity group. For the low-capacity group, the evoked N270 was predominantly restricted to the time window of 295–360 ms, whereas for the high-capacity group it ranged from about 260 ms to 380 ms. Therefore, the averaged amplitudes in the two time-windows were measured and put into the mixed ANOVA.

The mixed ANOVA revealed a significant main effect of Change-type [*F*(3,66) = 12.64, *p*<0.001, partial*η^2^* = 0.37, *ω* = 0.99], but no significant Group×Change-type interaction [*F*(3,66)<1, *p*>0.8, partial*η^2^* = 0.01, *ω* = 0.01], suggesting that the N270 was not modulated by the VWM capacity. There was no difference between the low-capacity and the high-capacity group on the amplitude of N270 [*F*(1,22)<1, *p*>0.8, partial*η^2^* = 0.01, *ω* = 0.05].

Further planned contrasts within each capacity group confirmed the non-significant Change-type×Group interaction. There was no difference between irrelevant change and no change in both low-capacity group [*F* (1,11)<1, *p*>0.30, partial*η^2^* = 0.07, *ω* = 0.13] and high-capacity group [*F* (1,11)<1, *p*>0.50, partial*η^2^* = 0.03, *ω* = 0.03]. In both capacity groups, the N270 amplitudes for relevant change and both change were significantly larger than that for no change [all *p*s<0.05, partial*η^2^*>0.3, *ω*>0.32]. No difference was found between the latter two conditions [*F*(1,11)<1].

#### Correlation

**Figure 6:**
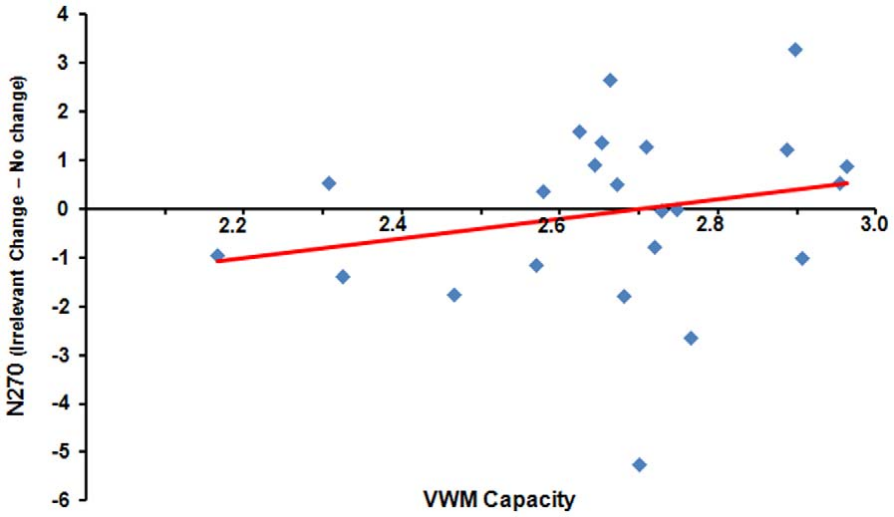
The correlation between individual VWM capacity and the corresponding N270 amplitude of the irrelevant change. The red line is the linear regression line fitting the data points.

Similar to the results showed in the irrelevant shape condition, the correlation between the VWM capacity and N270 amplitude was weak when the fine-grained feature served as the irrelevant dimension [Pearson *r* = 0.17, *p* = 0.43] (see Figure 6).

#### Interim Discussion

For the fine-grained orientation as the irrelevant feature, the overall results (behavioral and ERP) in our previous study [16] were replicated, suggesting that the irrelevant fine-grain information could not be selected into VWM. Moreover, behavioral, ERP, and correlation analysis provided converging evidence supporting the claim that this non-selection was not modulated by VWM capacity.

Although in Experiment 1, three pieces of evidence from different aspects (i.e., behavioral performance, ERP, and correlation) consistently suggested that the feature-based filtering was not modulated by the VWM capacity, the establishment of a null effect always calls for more explorations (e.g., using different condition, task, manipulation) since the possibility that there is an exception always exists. In addition, the non-influence of the VWM capacity may be because the participants' capacity was rather close. Indeed, the VWM capacity estimates in Experiment 1 were all in the range of 2∼3 objects (see Figure 4 and 6). The two VWM groups thereby may be not significantly distinguished from each other in nature, which might contaminate our conclusions. Experiment 2 was designed to rule out these possibilities.

## Experiment 2

As we stated in the introduction, ample evidences have demonstrated that the irrelevant high-discriminable information can be selected into VWM [16], [17], [20], [22], [23]. By taking these findings and the results of Experiment 1 into consideration, we tentatively suggested that only the irrelevant fine-grained information may have a chance to be modulated by VWM capacity, yet Experiment 1 failed to reveal. Therefore, Experiment 2 further explored the fate of the fine-grained information, by using Landolt-Cs of Experiment 1 as material, but in a *one-item* condition. If the participants could encode the irrelevant dimension, this condition should be the best condition since the VWM load was very low. In addition, we exerted a more strict control over the VWM capacity of the participants (see methods below).

### Methods

#### Participants

Twenty right-handed students (8 females; mean age 23.4 years) took part in the experiment. Ten of them had attended one of our previous VWM behavioral experiments requiring to remember 2 or 4 simple objects (which was different from the current Experiment 1 and 2) at least 2 months before and were naïve to the current experiment. Importantly, all these 10 participants had an averaged accuracy of below 75% in the previous VWM task. We considered that these participants may have a lower VWM capacity, since an averaged accuracy of 85% for remembering 2 or 4 simple objects was usually found in our previous VWM studies [9], [10], [17] and many other VWM studies [6]–[8]. All participants provided written and informed consent before experiments, and all procedures were approved by the Research Ethics Board of Zhejiang University.

#### Stimuli

Colored landolt-Cs (Figure 1A) used in Experiment 1 were adopted for the ERP experiment. In addition, a set of 6 colored-squares (1.01°×1.01° of visual angle), consisting of the first five colors of Experiment 1 and cyan (0, 255, 255), were adopted for measuring the VWM capacity.

#### Procedure and Design

The whole experiment included two sessions. The participants first completed a behavioral experiment to measure their VWM capacities. In this session, a memory set of 4 distinct color-squares were randomly displayed within a radius of 5° around fixation, with a constraint that the distance between two objects was at least 3.5° (center-to-center). The memory set was presented for 500 ms, followed by a 900 ms blank interval. Finally, a probe was presented at the center of the screen until a response. The participants were asked to judge whether it had appeared in the memory set. The accuracy was recorded for analysis.

After the completion of the behavioral experiment and had a rest of at least 15 minutes, the participants began the ERP experiment, in which only one colored landolt-C was presented at the center of the screen each time. The color of the object was the relevant feature while the orientation was the irrelevant feature. When there was a change on the irrelevant dimension, only one orientation was changed. The participants were asked to respond as accurately and quickly as possible to the probe (S2), which was displayed until a response was initiated or up to 2 seconds from its onset. Both response accuracy and RT were recorded and analyzed.

There were 40 trials in the behavioral session. As to the ERP session, participants completed 80 trials under each of the 4 conditions, resulting in a total of 320 trials which were presented randomly. The experiment was divided into 4 blocks with 5-minute break in between. The whole experiment lasted about 40 minutes in total.

#### Electrophysiological Recording and Analysis

EEG was recorded from 64 scalp sites using Ag/AgCl electrodes mounted in an elastic cap. The EEG and EOG were amplified by SynAmps2. Because more electrodes were available, we analyzed and averaged the following 15 electrodes to have a general match with the distribution of the electrodes analyzed in Experiment 1: FP1, FP2, FPZ, AF3, AF4, F3, F4, F1, F2, FZ, FC3, FC4, FC1, FC2, and FCZ. The ERP waveforms in these 15 electrodes were similar to each other. An average of 12.2% of trials was excluded from further ERP analysis.

The other aspects of the methods were identical to Experiment 1.

### Results and Discussion

#### VWM Capacity

We first estimated the VWM capacity by using Cowan's *K* formula for each participant. Then the participants were split into two groups (10 participants each) according to the median of K. The K ranged from 0.93 to 3.79 (see the result of Correlation), suggesting that the current experiment indeed had a better control over participants' VWM capacity. For the low-capacity group, the mean VWM capacity was about 1∼2 objects (K = 1.67), whereas for the high-capacity group, the mean VWM capacity was about 3∼4 objects (K = 3.06).

#### Behavioral Data

**Figure 7:**
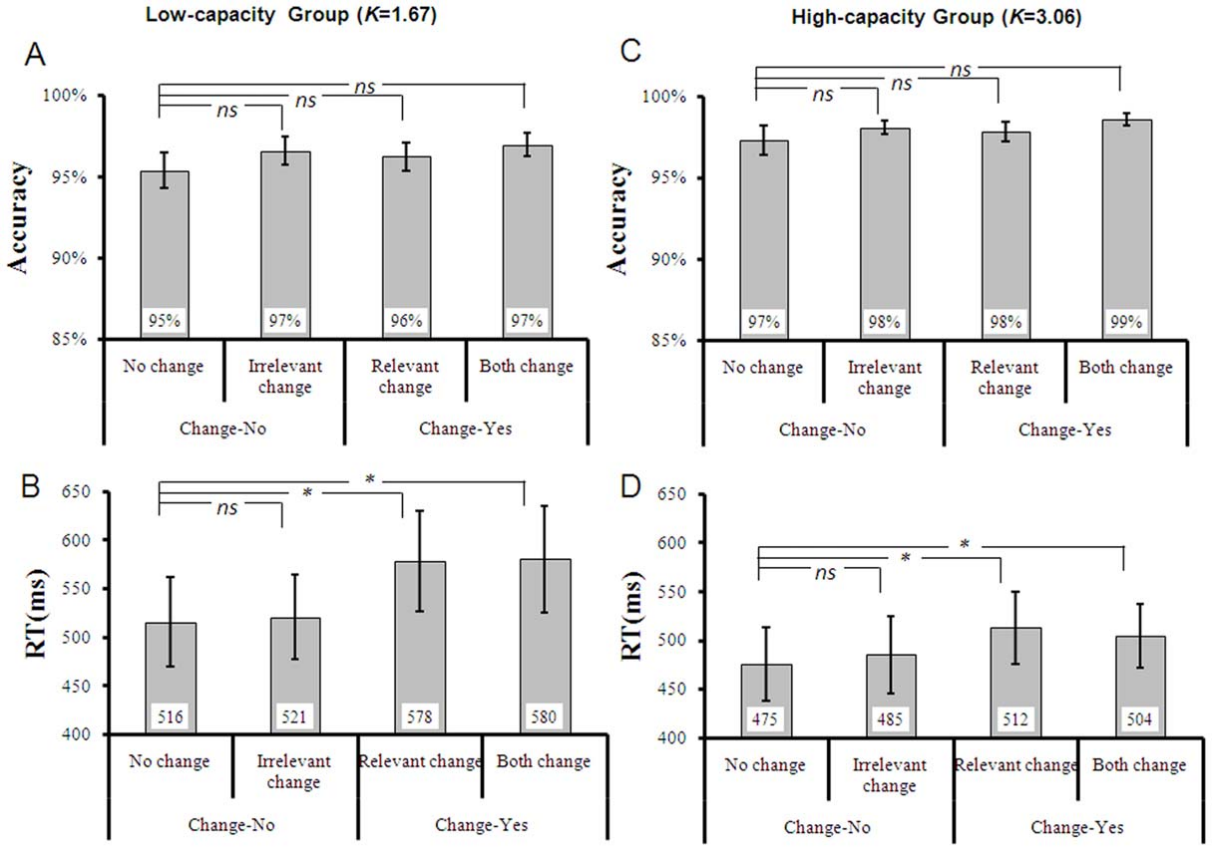
Behavioral results of Experiment 2. The left column shows the accuracy (A) and RT (B) of the low-capacity group. The right column shows the accuracy (D) and RT (D) of the high-capacity group.

The behavioral results were shown in Figure 7. For the accuracy, the mixed ANOVA revealed that the accuracy was significantly higher in the high-capacity group (98%) than in the low-capacity group [96%; *F*(1,18) = 5.67, *p*<0.05, partial*η^2^* = 0.24, *ω* = 0.62]. However, neither the main effect of Change-type [*F*(3,54) = 1.94, *p*>0.1, partial*η^2^* = 0. 10, *ω* = 0.47] nor its interaction with Group [*F*(3,54)<1, *p*>0.9, partial*η^2^* = 0, *ω* = 0.056] was significant. Planned contrast showed that there was no difference between the irrelevant change (97%) and no change [96%; *F*(1,18) = 3.61, *p*>0.05, partial*η^2^* = 0.16, *ω* = 0.43], suggesting that the irrelevant orientation was not encoded into VWM and interfered the detection of relevant colors. The other ones were also not significant, either [*p*s>0.05].

Further planned contrast within each capacity group confirmed the finding of Experiment 1 about the storage of irrelevant feature. There was no difference between irrelevant change and no change in either the low-capacity group (Figure 7A) [*F* (1,9) = 2.16, *p*>0.1, partial*η^2^* = 0.19, *ω* = 0.26] or the high-capacity group (Figure 7C) [*F* (1,9) = 1.45, *p*>0.2, partial*η^2^* = 0.14, *ω* = 0.19]. The other ones were also not significant [*p*s>0.1].

The mixed ANOVA on RT revealed that there was no significant difference between the low-capacity group (548 ms) and the high-capacity group [493 ms; *F*(1,18)<1, *p*>0.3, partial*η^2^* = 0.04, *ω* = 0.14]. However, the main effect of Change-type was significant [*F*(3,54) = 22.43, *p*<0.01, partial*η^2^* = 0.56, *ω* = 1.0], as well as its interaction with Group [*F*(3,54) = 3.46, *p*<0.05, partial*η^2^* = 0.16, *ω* = 0.77], suggesting that the Change-type affected the performance differently in the two groups. Planned contrast based on all the participants showed that there was no difference between irrelevant change (495 ms) and no change [502 ms; *F*(1,18) = 3.16, *p*>0.05, partial*η^2^* = 0.15, *ω* = 0.39], implying that the irrelevant orientation was not encoded into VWM. The RT of relevant change [545 ms; *F*(1,18) = 39.73, *p*<0.001, partial*η^2^* = 0.69, *ω* = 1.0] and both change [542 ms; *F*(1,18) = 28.82, *p*<0.001, partial*η^2^* = 0.62, *ω* = 1.0] were significantly longer than no change (502 ms). No difference was found between relevant change and both change [*F*<1].

We conducted a one-way ANOVA by taking Change-type as the factor within each capacity group to elaborate the significant Change-type×Group interaction. For the low-capacity group (Figure 7B), the main effect of Change-type was significant [*F* (3,27) = 15.04, *p*<0.001, partial*η^2^* = 0.63, *ω* = 1.0]. Planned contrast showed that there was no difference between irrelevant change and no change [*F* (1,9) = 1.07, *p*>0.1, partial*η^2^* = 0.11, *ω* = 0.15]. Yet relevant change [*F*(1,9) = 21.09, *p* = 0.001, partial*η^2^* = 0.70, *ω* = 0.98] and both change [*F*(1,9) = 18.01, *p*<0.01, partial*η^2^* = 0.67, *ω* = 0.96] yielded significantly slower responses than no change. No difference was found between relevant change and both change [*F*<1]. For the high-capacity group (Figure 7D), the main effect of Change-type was significant, too [*F*(3,27) = 8.01, *p* = 0.001, partial*η^2^* = 0.47, *ω* = 0.98]. Planned contrast showed that there was no difference between irrelevant change and no change [*F* (1,9) = 2.11 , *p*>0.1, partial*η^2^* = 0.19, *ω* = 0.26]. Relevant change [*F*(1,9) = 21.14, *p* = 0.001, partial*η^2^* = 0.70, *ω* = 0.98] and both change [*F*(1,9) = 12.15, *p*<0.01, partial*η^2^* = 0.58, *ω* = 0.87] caused significantly slower RTs than no change. No difference was found between relevant change and both change [*p*>0.1]. In sum, although there was a significant Change-type×Group interaction, it was not due to the different result profiles concerning the selection of the irrelevant feature.

#### ERP Data

**Figure 8:**
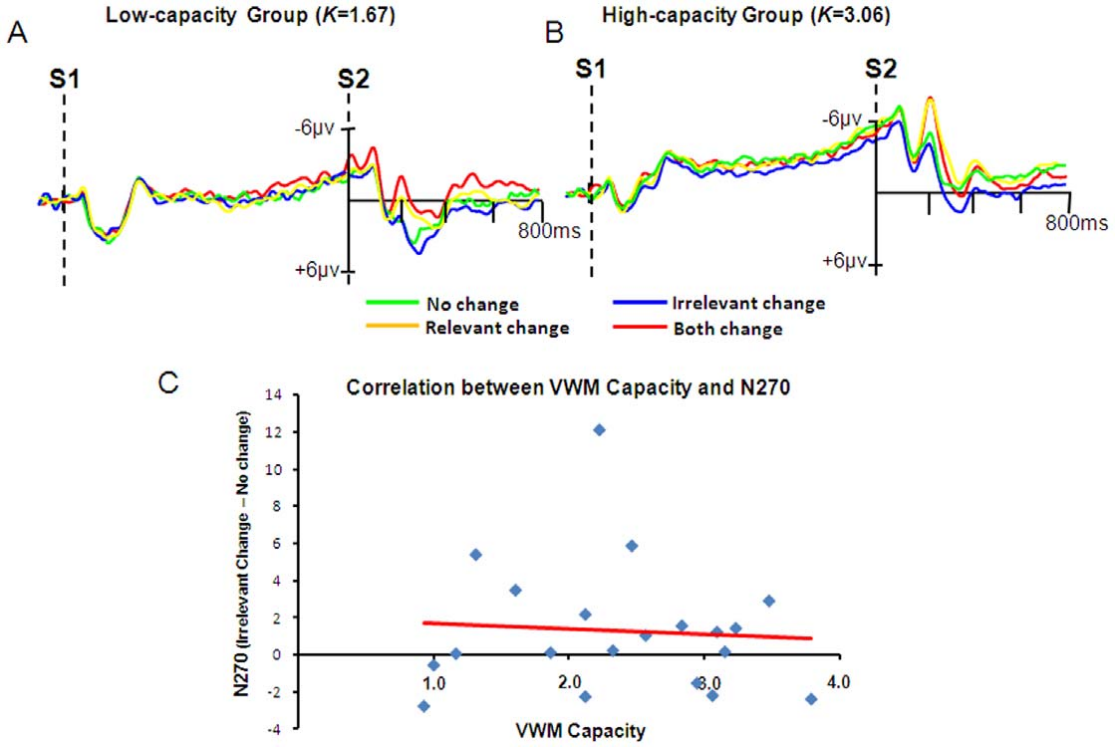
The ERP Results and correlation of Experiment 2. The top row (A) shows the ERP results for the low-capacity and the high-capacity group. The bottom row (B) shows the correlation between individual VWM capacity and the corresponding N270 amplitude of the irrelevant change. The red line is the linear regression line fitting the data points.

Replicating the results of Experiment 1 and our previous findings [16], the change of irrelevant fine-grained feature did not evoke N270 in both the low-capacity and the high-capacity groups (Figure 8A & B). As revealed in our previous study [16], the latency of N270 was earlier in the current experiment than in Experiment 1 for the current experiment was easier than Experiment 1. A time window of 200–290 ms was used in the mixed ANOVA to test the effect of Change-type and Group.

The ANOVA revealed a significant main effect of Change-type [*F*(3,54) = 12.04, *p*<0.001, partial*η^2^* = 0.40, *ω* = 0.99], yet its interaction with Group was not significant [*F*(3,66)<1, *p* = 0.90, partial*η^2^* = 0.01, *ω* = 0.08], suggesting that the N270 was not modulated by the VWM capacity. In addition, the amplitude was more negative in the high-capacity group than in the low-capacity group [*F*(1,18) = 9.34, *p*<0.01, partial*η^2^* = 0.34, *ω* = 0.82], which was due to the higher CNV (contingent negative variation) during S1 and S2 in the high-capacity group.

Further planned contrast showed that there was no difference between irrelevant change and no change in both the low-capacity group [*F* (1,11) = 1.22, *p* = 0.30, partial*η^2^* = 0.12, *ω* = 0.17] and the high-capacity group [*F* (1,11) = 1.05, *p*>0.30, partial*η^2^* = 0.11, *ω* = 0.15]. In both capacity groups, relevant change and both change yielded significantly more negative amplitude than no change [all *p*s<0.05, partial*η^2^*>0.35, *ω*>0.55]. No difference was found between the latter two [*F*(1,9)<1].

#### Correlation

Replicating the finding of Experiment 1, a non-significant correlation was revealed between the VWM capacity and irrelevant-change-related N270 amplitude (Figure 8C), Pearson *r* = −0.07 [*p*>0.70].

By examining a condition in which the irrelevant fine-grained information was most likely to be encoded and exerting a more strict control over the VWM capacity, we did not find any evidence on accuracy, RT, N270 or the correlation supporting the selection of the irrelevant fine-grained feature. Therefore, the null-effect of the VWM capacity on the feature-based filtering revealed in Experiment 1, to some extent, was fairly robust.

## Discussion

The goal of the current study was to explore whether the VWM capacity modulated the feature-based filtering of the task-irrelevant information in VWM. To achieve this goal, in two experiments we examined whether the filtering of the two types of task-irrelevant information (i.e., the high-discriminable feature versus the fine-grained feature) was different in the low-capacity and the high-capacity group. Contrast to the prediction that VWM capacity modulated the feature-based filtering, we replicated our previous findings in both the low-capacity and the high-capacity groups [16]. That is, the irrelevant high-discriminable information was extracted into VWM involuntarily, whereas the irrelevant fine-grained information was filtered out regardless of VWM capacity. Moreover, no significant correlation was found between the irrelevant-change-related N270 and the VWM capacity. These results suggest that the feature-based filtering is not modulated by VWM capacity.

Although the low VWM capacity participants have lowered ability in filtering out the distracting information presented in distinct spatial locations [12], [13], [14], the results of the current study suggest that there is no deficit for the low-capacity group in filtering out the task-irrelevant information which shares the same spatial location with the task-relevant information. This difference in filtering suggests that the mechanism for the feature-based filtering is different from that for the spatial-based filtering. It has been suggested that the reason for the filtering difference between the low-capacity and the high-capacity group in previous findings was because compared to the high-capacity group, the low-capacity group are slower in disengaging from the location of the distracters once the spatial-attention was attracted to it [14], [32], [33]. In contrast, for the materials used in the current study, the relevant and irrelevant information pertained to the same object. Therefore, it seems that if the irrelevant information is attended (i.e., high-discriminable information), the participants do not need to disengage the feature-based attention from the distracter dimension while keeping it on the target dimension. This may be attributed to the characteristics of the feature-based attention, in which each feature can be selected independently based on its own resource without competition.

We considered that the execution of the feature-based filtering at least was partially dependent upon the processing level of the task-irrelevant information required in perception. Recently we have provided ERP [16] and behavioral [17] evidences suggesting that only the irrelevant information which is processed at the parallel stage of perception could be selected into VWM automatically. The results from the current study provided further evidence supporting this view. Particularly, the current findings suggest that the involuntary selection of the high-discriminable information is not attributed to the low-capacity individuals, but a common phenomenon for all individuals (at least for the healthy adults). On the other hand, we also revealed that even for the low-capacity individuals, who had a relatively loose top-down control such that they may select the irrelevant information, neither the behavioral results nor the ERP results revealed a trace of being selected for the fine-grained information.

It is worth noting that the current results and previous findings on information selection in VWM together [22], [34] exhibit a great analogy to the findings in the perceptual load theory [35], [36]. Firstly, it has been revealed that although the perception load of a perceptual task modulates the information selection when the information is presented in distinct spatial locations, it does not modulate the information selection when both the relevant and irrelevant information pertain to the same object [37]. Second, the perceptual load theory predicts that for the relevant and irrelevant information displayed in different spatial locations, increasing the perceptual load of the relevant dimension will decrease the irrelevant processing [35], [36]. In concert with this prediction, a recent VWM study suggested that the involuntary selection of high-discriminable information may only fit for the low VWM load condition (e.g., remembering 2∼3 objects), but attenuated or vanished at the high VWM load (e.g., remembering 6 objects) [22], [34]. Considering these similarities, more work is worth doing to elaborate the interaction between the information selection in perception and that in VWM.

### Summary

In sum, we explored whether the VWM capacity modulated the feature-based filtering of the task-irrelevant information by adopting two types of visual information (high-discriminable information versus fine-grained information). The results revealed that regardless of the VWM capacity, high-discrimination information is always extracted into VWM yet fine-grained information is filtered out, suggesting that the VWM capacity does not modulate the feature-based filtering in VWM.
